# Anti-obesity effect of the bacterial product nisin in an NIH Swiss mouse model

**DOI:** 10.1186/s12944-023-01788-1

**Published:** 2023-02-10

**Authors:** M. K. Al-Emarah, H. R. Kazerani, F. Taghizad, H. Dehghani, M. Elahi

**Affiliations:** 1 Department of Basic Sciences, Faculty of Veterinary Medicine, Ferdowsi University of Mashhad, Mashhad, Iran; 2 Faculty of Agriculture and Marshlands, University of Thi-qar, Thi-qar, Iraq; 3 Stem Cell Biology and Regenerative Medicine Research Group, Research Institute of Biotechnology, Ferdowsi University of Mashhad, Mashhad, Iran

**Keywords:** Obesity, Nisin, Bacteriocin, Microbiota

## Abstract

Obesity is a life-threatening metabolic disorder that predisposes individuals to other diseases. In this study, the effect of nisin, a bacteriocin produced by some bacteria, on an animal model of obesity based on selected parameters was investigated. Forty Swiss NIH mice were randomly divided into four groups and received either a placebo (saline) or nisin (25, 50, or 100 μg/kg, ip) daily for 8 weeks. The mice in all groups were fed a high-sugar diet throughout the experiment. Bodyweight and food intake were measured weekly, and at the end of the experiment, the levels of FBS, serum triglyceride, cholesterol, high-density lipoprotein, low-density lipoprotein, and hepatic enzymes were tested, and red and white blood cell counts, hemoglobin concentration, mean corpuscular volume, mean corpuscular hemoglobin, and mean corpuscular hemoglobin concentration were determined. Finally, the expression levels of some obesity-related genes, including stearoyl-CoA desaturase-1 (SCD-1), glucose transporter-4 (GLUT4), zinc finger protein 423 (zfp423), 422 (ap2), and tumor necrosis factor-alpha (TNF-α), were assessed using reverse transcriptase-quantitative polymerase chain reaction (RT–qPCR). After the experiment, the body weights, abdominal fat, and body mass index were significantly lower in the nisin-treated groups than in the control group. The highest effect was observed with 50 μg/kg nisin. The expression of SCD-1, GLUT4, 422(ap2), and TNF-α decreased significantly following treatment with nisin. No significant differences were observed in the other studied parameters, and no toxic effects were observed for nisin under these experimental conditions. The results suggested that nisin could have antiobesity effects.

## Introduction

Obesity and overweight refer to the excessive accumulation of fat in various parts of the body. According to the World Health Organization (WHO), overweight is defined by a body mass index (BMI) between 25 and 30 kg/m^2^. When BMI exceeds 30 kg/m^2^, the condition is referred to as obesity. According to WHO reports, nearly four million people a year lose their lives as a consequence of the complications caused by obesity. Obesity and overweight were once considered to be problems of wealthy societies; however, currently, more people in intermediate and poor societies are dealing with this phenomenon [1].

Obesity can lead to a variety of health issues ranging from heart conditions to hypertension and even cancer. In addition to an increased burden on the skeletal system, particularly the joints, which is a natural consequence of excessive weight, the production of interleukin-6 (IL-6) from fat cells predisposes the individual with obesity to a state of chronic mild inflammation. The secretion of fatty acids from the adipose tissue, which finally lodge in the muscular and liver tissues, is a preliminary stage of developing insulin resistance and diabetes. Moreover, estrogens produced by enlarged adipocytes could lead to a proliferative and eventually cancerous state in the breast [2].

Overweight and obesity occur when the rate of energy supply exceeds that of consumption over a period of time long enough to allow the accumulation of fat droplets in adipocytes. Different causes have been suggested for this process, mostly related to diet and physical activity patterns. However, the effects of diet and physical activity can be augmented or attenuated by genetic factors and the metabolic state, as seen in some genetic disorders causing obesity [3]. There is an escalating interest in developing new treatments for obesity and its related complications. Some drugs have succeeded in obtaining FDA approval in this regard. However, there are some side effects associated with these treatments. Other herbal medications have also been introduced, but there are information gaps that should be filled with further studies [4–7].

*The microbiota* refers to the microbial population of different organs throughout the body. These microorganisms might include bacteria, fungi, and viruses. The most studied role of *the microbiota* in human health is in relation to the gut microbiota and particularly the resident bacteria. The bacterial population of a healthy gut mostly comprises two phyla: Bacteroidetes and Firmicutes. These phyla and others play crucial roles in the maintenance of intestinal integrity, immunomodulation, protection against pathogens, and drug metabolism [8]. Several studies have shown the relationship between the composition of the microbial population in the digestive system and body composition/weight [9]. A study in children showed that children affected by obesity had a lower abundance of bacterial species in the intestines [10]. The same observation has been verified in adults [11].

Bacteriocins, peptides produced and secreted by certain strains of bacteria, appear to have significant effects on the regulation of weight. A bioinformatics study suggests that certain bacteriocins of the family Lactobacillaceae, which are probiotics of the human intestine, could exert some effects on weight and adiposity [12]. One recent study using the bacteriocin gassericin A from the probiotic *L. gasseri* LA39 on 3T3-L1 preadipocytes showed a significant reduction in the expression of some genes related to adipogenesis [13].

Nisin, a bacteriocin discovered by Rogers and Whittier in 1928, is a 3500-Da peptide containing 34 amino acids and has bactericidal properties. Nisin is produced during the exponential growth phase of *Lactobacillus lactis,* subspecies *lactis*; when the growth of bacteria has halted, the production of the peptide also halts [14]. Nisin has long been used in the food industry as a preservative, and it is produced on a commercial scale to be used in packed foods. Nisin has shown a strong inhibitory effect toward gram-negative bacteria [15]. Accordingly, it was decided to conduct an experiment using this bacteriocin to evaluate its effects on weight, some serum factors, and some significant genes involved in the development and growth of adipocytes.

## Materials and methods

Nisin was purchased in the form of balanced 2.5% sodium chloride lyophilized powder (Sigma–Aldrich, Germany) and was diluted with normal saline containing 0.05% acetic acid.

### Animals and treatment

Forty healthy male NIH Swiss mice in their 6th week of life weighing 25–45 g were chosen for the experiment. The mice were then acclimatized for 2 weeks before the research. The animals were treated according to the guidelines of the Animal Ethics Committee of the Ferdowsi University of Mashhad (IR.UM.REC.1400.250). The mice were kept in individual cages throughout the experiment. The mice were randomly divided into four groups of ten: the control group that received a placebo and the test groups that received nisin at 25, 50, and 100 μg/kg daily (single dose) for 8 weeks. The mice were fed ad libitum on a high-sugar diet beginning 5 days before the start of the experiment (Table 1). To prepare the diet, 450 g of sugar was dissolved in 100 ml of distilled water at 100 °C. The hot thick syrup was homogenously mixed with 450 g of commercially available mouse food (Javaneh Khorasan Co., Mashhad). It was then allowed to dry at room temperature. At the end of each week, the body weights and the amount of consumed food per animal were measured.

**Table 1:** Nutrition information of the diet fed to the mice during the experiment

Nutrients	Quantity
Energy (kcal/kg)	3345
Protein (%)	10–10.5
Fat (%)	1–1.5
Fiber (%)	2.5–3
Sucrose (%)	50
Methionine (%)	0.025
Lysine (%)	0.025
Salt (%)	0.25
P/Ca ratio	1.5–2.5
Ash (%)	2

### Sampling

At the end of the 8th week, the mice were deprived of food for 12 hours and anesthetized with 50 mg/kg thiopental sodium, and their mesenteric fat was extracted and kept in liquid nitrogen for further molecular tests. Blood sampling was performed from the abdominal vena cava. Blood serum was separated by centrifugation at 3500 rpm, and the serum was kept at − 20 °C for further biochemical assays. The weights of the kidneys and spleen, the length of the small intestine, and the BMI (body weight in kilograms/nasoanal length in metre^^2^) were measured.

Serum parameters, including glucose, cholesterol, triglyceride, high-density lipoprotein (HDL), low-density lipoprotein (LDL), alanine transaminase (ALT), aspartate transaminase (AST), and alkaline phosphatase (ALP), were measured using enzymatic colorimetric methods (kits: Pars Azmoon®, Iran; system: Autoanalyzer Alpha Classic®). Differential blood cell count and the measurements related to hemoglobin were performed by the partial defeat refresh method (Sysmex-XP-100).

### RNA isolation and gene expression

Total RNA was extracted from mesenteric adipose tissue using the Total RNA Extraction Kit (DENAzist Asia, Iran). To check the effect of nisin on the level of expression of adipocyte-specific genes, four genes were assayed using reverse transcriptase-quantitative polymerase chain reaction (RT–qPCR): stearoyl-CoA desaturase-1 (SCD-1) gene, which regulates a rate-limiting step in the production of unsaturated fatty acids, SLC2A4 gene encoding glucose transporter-4 (GLUT4) as the main transporter of glucose into the adipocytes, zinc finger protein 423 (zfp423) gene encoding zinc finger protein 423 as a determinant of differentiation and maintenance of white adipocytes, and the gene encoding 422(ap2) protein as a fatty acid binding protein, which increases with the differentiation of preadipocytes to adipocytes. The expression of the tumor necrosis factor-alpha (TNF-α) gene was also assayed to evaluate any possible inflammatory response. To reduce the effect of any possible DNA contamination on the results of RT–qPCR, the primers were designed in exon–exon junction form. TATA-binding protein (TBP) was chosen as the reference (housekeeping) gene due to its higher stability in adipocytes [16]. The list and sequence of primers are shown in Table 2.

**Table 2:** The primers used in RT–qPCR

Gene	Forward primer	Reverse primer
422ap2	TGAAATCACCGCAGACGACA	ACACATTCCACCACCAGCTT
GLUT4	GCCCGGACCCTATACCCTAT	GGGTTCCCCATCGTCAGAG
SCD-1	CAGGTTTCCAAGCGCAGTTC	ACTGGAGATCTCTTGGAGCA
TBP	CCTATCACTCCTGCCACACC	ATGACTGCAGCAAATCGCTTG
TNF-α	TAGCCCACGTCGTAGCAAAC	GCAGCCTTGTCCCTTGAAGA
Zfp-423	CCGCGATCGGTGAAAGTTGA	ACGCTGTTCCTGTCTTCCAG

### Statistical analysis

Statistical analysis and drawing of the graphs were performed using GraphPad Prism Software version 9.4.1 (GraphPad Software Inc., USA). The nonparametric Mann–Whitney test was used for RT–PCR results. Comparisons of the body weights were carried out using two-way analysis of variance followed by the two-stage setup method of Benjamini, Krieger, and Yekutieli. All other comparisons were made using one-way analysis of variance followed by Dunnett’s multiple comparison test. *P* values less than 0.05 were considered statistically significant. Unless otherwise mentioned, all data are represented as the mean ± SEM.

## Results

### Body weight, total food intake, mesenteric fat, and BMI

The statistical analyses showed a significant decrease in weight in the treatment groups in comparison to the control group. As shown in Table 3, all three groups showed a significant loss of weight, with the greatest effect observed in group 3.

**Table 3:** The weekly body weights of the mice in different experimental groups

Nisin (μg/kg)	Body Weith (g)								
	Day 0	Day 7	Day 14	Day 21	Day 28	Day 35	Day 42	Day 49	Day 56
**0**	36.8 ± 1.3	39.6 ± 1.2	39.8 ± 1.4	39.6 ± 1.4	40.1 ± 1.3	40.2 ± 1.3	40.4 ± 1.4	40.8 ± 1.3	41.7 ± 1.5
**25**	34.7 ± 2.2	37.3 ± 1.9	37.3 ± 1.8	36.6 ± 1.7	37.0 ± 1.8	37.5 ± 1.7	37.6 ± 1.7	36.2 ± 1.6*	35.5 ± 1.5*
**50**	34.9 ± 1.2	37.1 ± 1.5	36.4 ± 1.2	35.6 ± 1.1	34.7 ± 1.4	35.6 ± 1.4	35.3 ± 1.5*	35.0 ± 1.5**	34.1 ± 1.5**
**100**	34.5 ± 2.1	37.2 ± 1.9	37.4 ± 2	38.0 ± 2	37.7 ± 1.9	38.1 ± 1.8	37.5 ± 1.5	36.6 ± 1.4*	36.0 ± 1.5*

All groups of mice were fed a high-sugar diet and were treated daily with nisin (25, 50, or 100 μg/kg, i.p.) or placebo

Data are represented as the mean ± SEM. *P < 0.05, **P < 0.01 vs. the control group

**Fig. 1:**
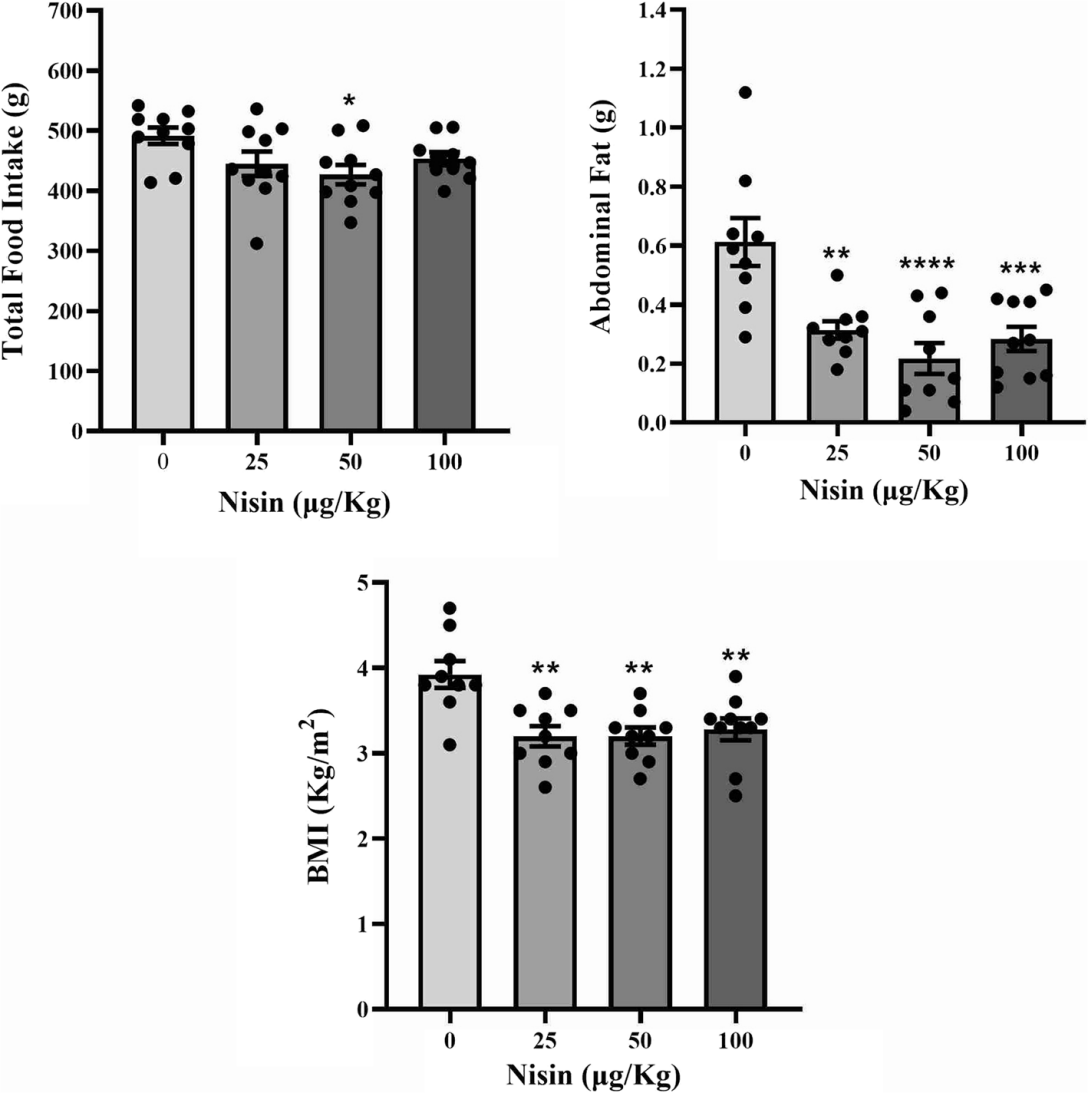
Total food intake, abdominal fat, and body mass index (BMI) of the mice. The mice had been treated with placebo or different doses of nisin (25, 50, or 100 μg/kg, i.p.), daily for 8 weeks. Data are shown as the mean ± SEM. The asterisks indicate statistical significance compared to the control group (*P < 0.05, **P < 0.01, ***P < 0.001, ****P < 0.0001)

The amount of food intake was only reduced in group 3 (50 μg/kg nisin), with other groups showing no significant changes (Fig. 1).

A much more significant change was seen in the amount of abdominal fat content, which showed a very sharp reduction in all three treatment groups, with group 3 showing the greatest effect (Fig. 1).

Another prominent effect of nisin was the change in BMI, which was significantly reduced in all three treatment groups (Fig. 1).

### Preliminary histological evaluation

The preliminary results regarding the cytometry of abdominal fat showed a prominent decrease in the size of adipocytes in the test groups. However, the minute number of specimens in nisin-treated animals did not allow a sample size large enough for statistical comparison.

### PCR results

**Fig. 2:**
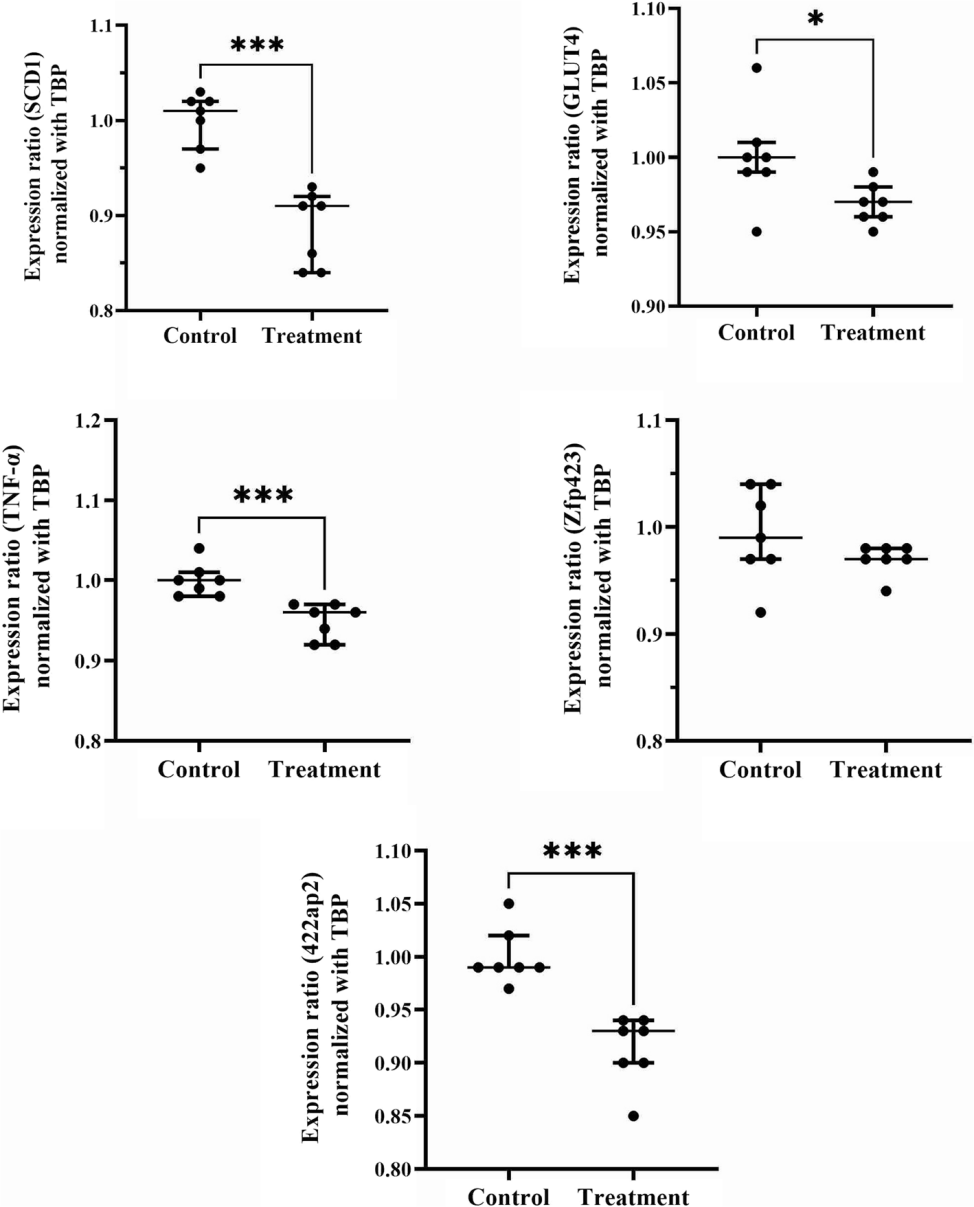
The level of gene expression in mesenteric fat of the experimental animals. The mice had been treated with either a placebo (the control) or nisin (50 μg/kg, i.p.) for 8 weeks daily. Individual data are shown together with median and quartiles. SCD-1: stearoyl-CoA desaturase-1, TBP: TATA-binding protein, GLUT4: glucose transporter-4, TNF-α: tumor necrosis factor-alpha, zfp423: zinc finger protein-423. *P < 0.05, ***P < 0.001

The RT–qPCR data showed that the expression levels of SCD-1, GLUT4, TNF-α, and 422ap2 were significantly reduced, while that of zfp423 did not show any significant changes (Fig. 2).

### Organs, markers of blood, and serum

The studied serum markers (including fasting blood sugar [FBS], cholesterol, triglyceride, HDL, LDL, AST, ALT, and ALP), the length of the small intestine, the weight of the kidneys and spleen, the red and white blood cell counts, the concentration of hemoglobin, packed cell volume, mean corpuscular volume (MCV), mean corpuscular hemoglobin (MCH), and mean corpuscular hemoglobin concentration (MCHC) did not show any significant changes (Table 4).

**Table 4:** The studied markers of serum, blood, and body organs. The mice received nisin (25, 50, or 100 μg/kg, i.p.) or placebo daily for 8 weeks. Blood or serum biomarkers and the weights of body organs were measured at the end of the experiment

Nisin (μg/kg)	FBS (mg/dL)	Cholesterol (mg/dL)	Triglyceride (mg/dL)	HDL (mg/dL)	LDL (mg/dL)	AST (U/L)	ALT (IU/L)	ALP (IU/L)	RBC (× 10^**6**^)	WBC (× 10^**3**^)	Hb (g/dL)	PCV (%)	MCV (fL)	MCH (pg)	MCHC (g/dL)	Small Int (cm)	Kidney Wt (g)	Spleen Wt (g)
**0**	93 ± 6.4	116 ± 11.5	84.7 ± 14.6	48.6 ± 5	53 ± 5.1	369 ± 48.2	99.9 ± 10.6	176 ± 24	8.09 ± 0.357	5.16 ± 0.78	12.2 ± 0.6	40.1 ± 1.8	49.5 ± 0.6	15.1 ± 0.2	30.5 ± 0.3	49.9 ± 1.9	0.61 ± 0.02	0.27 ± 0.03
**25**	90.1 ± 3.6	113 ± 10.3	75.6 ± 8.9	46.2 ± 4.2	51.7 ± 4	317 ± 59.6	118 ± 25.9	182 ± 13.8	7.74 ± 0.244	4.98 ± 0.76	11.6 ± 0.3	38.6 ± 0.9	50.1 ± 1.4	15.1 ± 0.2	30.2 ± 0.5	49.6 ± 1.5	0.6 ± 0.04	0.31 ± 0.05
**50**	77.7 ± 9.8	128 ± 8.6	90.4 ± 12.1	50.4 ± 3.9	57 ± 4	412 ± 67.6	112 ± 8.5	163 ± 16.8	8.49 ± 0.418	6.76 ± 1.2	12.8 ± 0.7	42.1 ± 2.3	49.6 ± 1	15.1 ± 0.5	30.4 ± 0.5	46.2 ± 1.6	0.61 ± 0.04	0.4 ± 0.04
**100**	92.3 ± 3.9	114 ± 12.7	87 ± 6.5	49.1 ± 2.6	56.2 ± 3	465 ± 55	140 ± 26.7	175 ± 18.8	8.21 ± 0.565	5.99 ± 0.48	12.3 ± 0.8	42.1 ± 2.4	52 ± 1.6	15.1 ± 0.3	29 ± 0.4	48.2 ± 1.3	0.64 ± 0.04	0.38 ± 0.06

All data are expressed as the mean ± SEM.

*FBS* fasting blood sugar, *HDL* High-density lipoprotein, *LDL* Low-density lipoprotein, *AST* Aspartate transaminase, *ALT* Alanine transaminase, *ALP* Alkaline phosphatase, *RBC* Red blood cell, *WBC* White blood cell, *Hb* Hemoglobin, *PCV* Packed cell volume, *MCV* Mean corpuscular volume, *MCH* Mean corpuscular hemoglobin, *MCHC* Mean corpuscular hemoglobin concentration

**Fig. 3:**
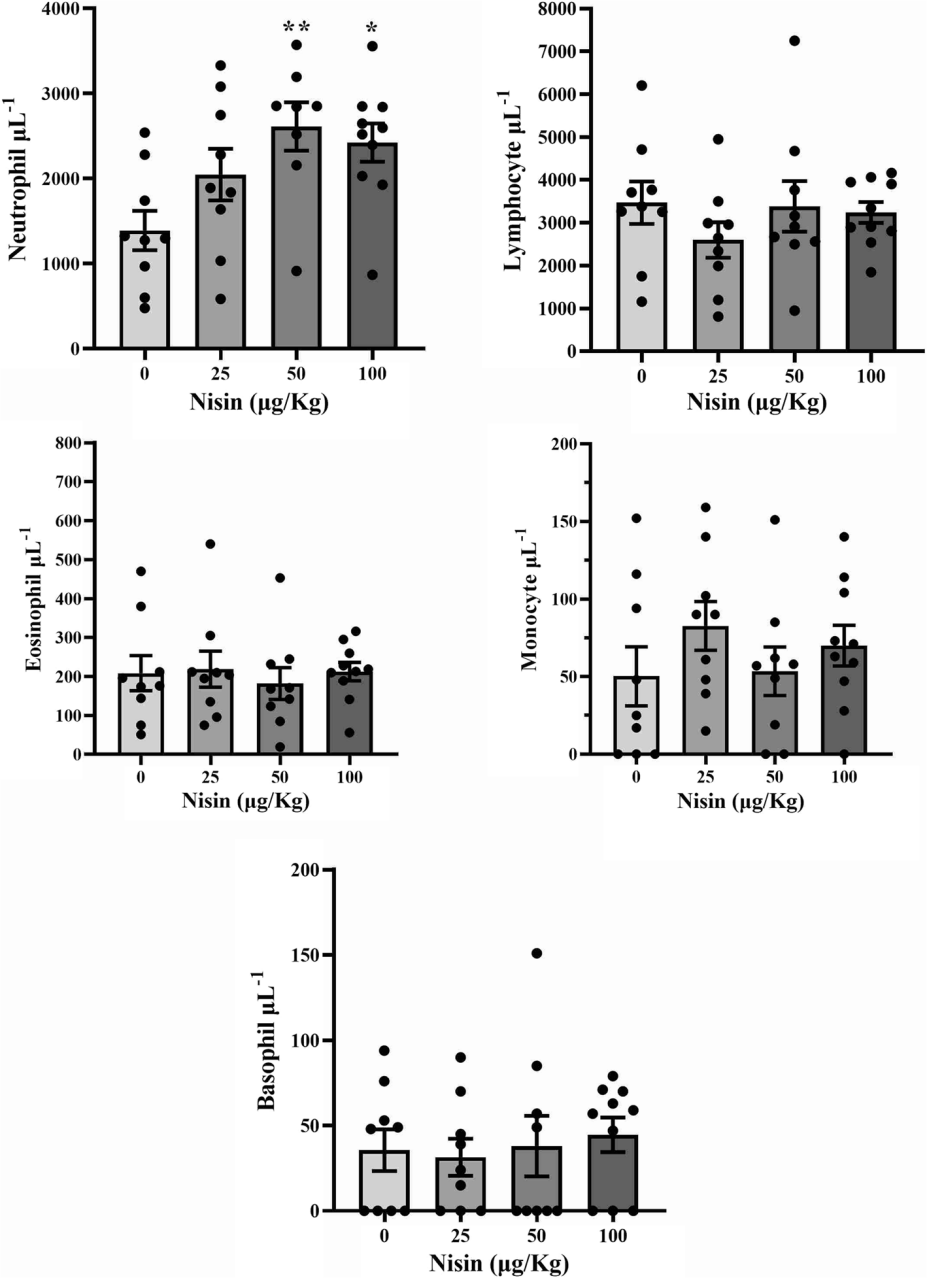
Differential count of white blood cells in different experimental groups. The mice in the test groups were treated daily with different doses of nisin (25, 50, or 100 μg/kg, i.p.) or a placebo (the control) for 8 weeks. Blood was sampled for the test at the end of the experiment. Data are presented as the mean ± SEM. *P < 0.05, **P < 0.01 vs. the control group

The results of differential white blood cell counts showed a significant increase in the number of neutrophils in the group receiving 50 and 100 μg/kg nisin. Other comparisons, however, did not yield statistical significance (Fig. 3).

## Discussion

Previous studies on bacteriocins have mainly focused on their antibacterial properties and their potential application as food preservatives. For instance, the antibacterial properties of nisin have been widely studied, and it has been extensively used alone or in combination with other substances as a food preservative [17]. Accordingly, some studies have evaluated the potential toxic effects of nisin, but only the oral route of administration has been tested, and these studies have mainly supported the safety of this peptide as a food preservative, which led to its approval by the Food and Drug Administration [18, 19]. However, there is no information regarding the potential therapeutic effect of nisin against obesity. This team first conducted a pilot study using nisin in laboratory rats, and the results showed a significant decrease in the adipose content in the abdominal area (unpublished results). Therefore, it was decided to use nisin in this study to evaluate its possible anti-obesity activity.

The results suggested significant weight loss in animals treated with nisin via i.p. injection. A significant reduction in food intake was seen only in one of the treatment groups, so the effect may be accidental. However, if this effect is confirmed by future experiments, it can be considered one of the mechanisms by which nisin exerts its effect on weight reduction. A similar result of decreased food intake was seen in a study determining the biological activity of nisin as a food additive [20].

In this study, nisin caused a dramatic decline in the level of SCD-1 expression. Stearoyl CoA desaturase-1 is a rate-limiting enzyme in the production of palmitoleic and oleic acids, two important monounsaturated fatty acids that are produced in the liver. The gene for SCD-1 is suppressed by leptin, and the suppression of SCD-1 can lead to a considerable loss of weight in people affected by obesity [21]. A study in high-fat diet-fed rats showed that obesity-prone rats had a higher proportion of the SCD-1 product palmitoleic acid [22]. Consistently, in the present study, the body weight, abdominal fat content, and BMI were significantly lower in the animals that received nisin. It is noteworthy that the average BMI for mice aged 9–21 weeks has been reported to range from 3.52 ± 0.11 to 3.57 ± 0.02 g/cm^2^ [23], which is well below that of the control mice fed a high-sugar diet in the present study (3.92 ± 0.16 g/cm^2^). These findings suggest that nisin could cause a significant reduction in adiposity and body weight in vivo. However, treatment with nisin did not affect the concentrations of serum triglyceride and cholesterol, suggesting that the most noticeable effect of nisin is on the fat tissue, rather than serum factors. According to the present results, nisin might exert its weight-protecting effect, at least partly, by reducing food intake. It is noteworthy that the high level of SCD-1 expression and subsequently higher levels of monounsaturated fatty acids have also been related to higher cancer death in patients affected by obesity [24], conferring more benefits to the use of nisin as a health-protective agent.

In this study, treatment with nisin caused a significant reduction in the expression of the GLUT4 gene, which encodes the main transporter of glucose into adipocytes and skeletal muscle cells. The expression of GLUT4 is reduced in adipocytes (but not skeletal muscle cells) in type II diabetes mellitus (T2D) [25]. Impairment in the uptake of glucose by GLUT4 is the primary stage in the onset of T2D. Although the expression of GLUT4 was reduced in the animals, no impairment was observed in FBS, suggesting that at least in a period of 8 weeks, nisin did not increase the risk of diabetes.

Inflammation is a key element in the onset and development of insulin resistance in T2D. To evaluate the effect of nisin on the inflammatory response of the adipose tissue, the level of TNF-α expression in the adipose samples was assayed. It is generally accepted that the serum level of TNF-α is mildly increased in people affected by obesity. Moreover, the adipose tissue obtained from people affected by obesity had higher levels of TNF-α mRNA. On the other hand, increased levels of TNF-α reduce the uptake of glucose via GLUT4 by adipocytes [26]. In addition, TNF-α stimulates hepatocyte death and the progression of fatty liver disease. A reduction in the inflammation of adipose tissue in nonalcoholic fatty liver disease (NAFLD) improves the condition of the liver [27]. In the present study, nisin caused a significant and dramatic reduction in the level of expression of TNF-α in adipose tissue and thus seems to be beneficial from this point of view. However, more experiments are necessary to further evaluate this effect.

Zinc finger protein-423 is a key regulator in the differentiation and maturity of preadipocytes. The expression of this protein in preadipocytes leads to an increase in the cellular amounts of peroxisome proliferator-activated receptor gamma (PARγ), which can lead to the differentiation of adipocytes [28]. Deletion of zfp423 predisposes white adipocytes to enter the path of beige fat cells, which can reverse diet-induced obesity. The thermogenic program initiator in beige adipocytes, early B-cell factor 2 (Ebf2), is suppressed by the activation of zfp423, which leads to the maintenance of white adipocyte destiny [29]. Nisin did not significantly change the level of expression of zfp423 in this experiment. This might be an indicator that nisin probably does not have any significant effects on the fate of adipocytes but just reduces the amount of fat accumulation in these cells by repressing SCD-1 and probably some other yet-unknown mechanisms.

Adipocyte protein 422ap2 is expressed after the induction of adipose-specific genes CCAAT/enhancer binding protein (C/EBP) β, C/EBPα, and PPARγ. This protein is representative of adipocytes [30]. The expression level of 422ap2 was dramatically reduced in the cells treated with nisin, which is an indicator of reduced adiposity. Generally, since 422ap2 is not a fate determiner, it may be concluded that adipose tissue maintains its identity as a tissue; however, its capacity for producing and storing fat is reduced.

In this study, treatment with nisin increased the number of blood neutrophils but had no significant effects on other white blood cells. Neutrophilic leukocytosis, which is an increase in the number of circulating neutrophils, may be triggered by the introduction of bacterial products such as peptides into the bloodstream [31]. Since nisin is a bacterial peptide, such an increase was predictable.

A preliminary study on some potential side effects of parenterally injected nisin on the animals was also performed. Regarding the lack of previous studies, some routine screening tests, such as complete blood count, FBS, and the serum levels of triglyceride, cholesterol, HDL, LDL, and hepatic enzymes, were performed to monitor the general health status of the mice. The results of the tests showed no negative side effects. The blood profile of the animals was not different from that of the control group except for the increase in neutrophils. It is noteworthy that previous reports on the possible adverse effects of nisin as a food supplement are conflicting. While some studies report no adverse effect for supplementary nisin [20, 32], there are reports indicating weight loss and decreased food intake in laboratory animals [19]. Since there is no information regarding the bioavailability of nisin and whether it is absorbed from the intestine, the results of these studies may not be extrapolatable to the present study in which nisin was parenterally administered to the animals.

Diabetes mellitus is a destructive metabolic condition characterized by constant elevation of blood glucose levels, and FBS is a significant marker in predicting diabetes, even more reliable than hemoglobin A1c (HbA1c) [33]. No significant changes in the FBS level following 8 weeks of treatment with nisin were observed, suggesting no deteriorating effects on the ability of the body to manage glucose levels.

One of the major causes of death in modern societies is atherosclerosis, and high serum cholesterol is one of the main risk factors in this regard [34]. In addition to cardiovascular diseases, an increased cholesterol level has been associated with Parkinson’s disease [35] and some neural conditions, such as cerebral amyloidosis [36]. Nisin caused no changes in the levels of serum cholesterol, suggesting no related harmful effects under the studied conditions.

High levels of triglycerides, low levels of HDL, and high levels of LDL are indicators of potential cardiovascular risk [37]. There is also some evidence of a connection between the levels of these serum markers and the risk of some types of cancer [38]. Nisin did not cause any significant changes in the serum concentrations of these factors in this experiment.

One of the most important safety concerns of any therapeutic agent is its liver toxicity. The two enzymes AST and ALT are used to determine damage to the liver [39]. The levels of either enzyme did not change, denoting that nisin may not be hepatotoxic.

The red and white blood cell counts, the concentration of hemoglobin, and the factors denoting anemia (such as MCV and MCH) all remained normal during the experiment, suggesting that nisin may not exert negative effects on the general health condition.

### Comparisons with other studies: what does the current work add to the existing knowledge

Few studies have directly applied a purified bacteriocin to evaluate its effects on obesity. This study suggests that the bacteriocin produced by the probiotic *Lactobacillus lactis* may have important anti-obesity effects. There are a few studies regarding the anti-obesity effects of other bacteriocins [13, 40]; however, to the best of our knowledge, there is no such report on nisin.

### Study strengths and limitations

The direct implication of bacteriocins to evaluate their effects on obesity has been rare. Bacteriocins are hard to purify, and few bacteriocins are available commercially to be used in large amounts. This research is among the few studies in which a purified bacteriocin has been used to treat obesity. Although nisin showed some effects on the expression levels of some important genes related to fat synthesis, the lack of previous studies is one of the main limitations of the present study. For instance, the pharmacokinetics of nisin are not clear, and further studies are required to elucidate its potential intestinal absorbance, half-life, possible biodegradation, elimination route, etc. In this research, only a few doses of nisin were studied, and RT–PCR was performed for only one of the treatment groups, whereas more doses are necessary to be evaluated. Furthermore, histological studies were not possible because of the scarce amount of deposited fat in the test groups. In fact, this study was a preliminary study in this field, and it is necessary to study the potential anti-obesity effects of nisin in animals fed a high-fat/high-sugar diet. Obviously, employing an extra control group fed a normal diet provides better comparisons. Moreover, the assessment of other important genes, such as PPARγ, and the detection of downstream proteins are among other shortcomings that should be addressed in future studies.

## Conclusion

Great attention has been given paid to the biological effects and clinical importance of the intestinal microbiota in recent decades. It is too early to come to a concrete conclusion, but the same may be true regarding the bacteriocins produced by these microorganisms. Once the information gaps are filled with future studies, these peptides may have therapeutic or preventive applications for obesity and its related complications. Although therapy could never be a surrogate for a healthy lifestyle, paying attention to the human microbiota and its products is a factor not to be underestimated in dealing with this problem.

## Abbreviations

ALT: Alanine transaminase
ALP: Alkaline phosphatase
AST: Aspartate transaminase
BMI: Body mass index
FBS: Fasting blood sugar
GLUT4: Glucose transporter-4
HbA1c: Hemoglobin A1c
HDL: High-density lipoprotein
IL-6: Interleukin-6
LDL: Low-density lipoprotein
MCH: Mean corpuscular hemoglobin
MCHC: Mean corpuscular hemoglobin concentration
MCV: Mean corpuscular volume
NAFLD: Nonalcoholic fatty liver disease
PARγ: Peroxisome proliferator-activated receptor gamma
RT–qPCR: Reverse transcriptase-quantitative polymerase chain reaction
SCD-1: Stearoyl-CoA desaturase-1
TBP: TATA-binding protein
TNF-α: Tumor necrosis factor-alpha
T2D: Type II diabetes mellitus
WHO: World Health Organization
zfp423: Zinc finger protein-423
